# The History of Dentistry as a Co-ordinate Branch of the Healing Art

**Published:** 1891-06

**Authors:** F. S. Whitslar

**Affiliations:** Youngstown


### THE DENTAL REGISTER.

VOL. XLV.] JUNE, 1891. [No. 6.

### COMMUNICATIONS.

### The History of Dentistry as a Co-ordinate Branch of the Healing Art.

BY F. S. WHITSLAR, YOUNGSTOWN.

Presidential Address before the Northern Ohio Dental Association.

Every thought, every impulse, every principle or rule of action as well as every living thing has originated in a germ or its type. The being who rules in the heavens and among the inhabitants of this lower sphere, has in but few instances brought anything at once into mature existence. To be born, to grow, to arrive at maturity, to die, and to decay is but the history of every living thing as well as every work of creation. Institutions of learning, the sciences and arts, have been progressive in their character. It is not my purpose to trespass upon your time by giving you an elaborate history of dental science, but will concisely sketch the history of the dental art with a view of placing before you our position as a co-ordinate branch of the healing art. To do this it will not be necessary to determine the exact date at which the practice of medicine was first known.

Like many arts, its origin is involved in considerable obscurity; but this we know, that at a very early age the power to heal the sick, mitigate the pangs of suffering humanity, and stand between disease and death, was esteemed to be a God-given attribute. Much has been said and written respecting the antiquity of medicine. Now I claim the same antiquity for the dental art. The Ancients, who inclined to mythological rather than to natural causes, affirmed the science of medicine to be a divine emanation and impersonated it, first in Apollo and next in Esculapius. Thus its early history is mixed up with mythology and poetry.

Now while it is almost impossible to imagine a state of primitive society so happy as to be free from pain and disease, yet we find in proportion as people advanced towards civilization, and abandoned their more simple habits of living for idleness and luxury, so did disease in its various forms call into requisition the skill of the physician. Those same habits of refinement and luxury, and consequent attention to personal appearance must have rendered the practice of the dental art one of considerable importance even at a very early period of the world's history.

According to Heroditus there was a subdivision of medical science, and no practitioner was allowed to practice any but his own peculiar branch. Hence some were oculists, others attended solely to diseases of the head, others again to those of the teeth. It is not, however, till the time of Hippocrates, that we meet with any distinct notices of the diseases of the teeth. This appears the more extraordinary, as the significance of these organs, to say nothing of their useful or ornamental functions, was regarded by the ancient Egyptians in a very remarkable manner.

One of their most severe and degrading punishments consisted in the abstraction of a front tooth.

There can therefore be, I think, but little doubt that the manufacture of artificial teeth and other branches of the dental art were practiced at a much earlier date than that of which we have the first mention in history. The loss of the front tooth, whether by disease or otherwise, would, during the existence of that Egyptian punishment, give rise to unpleasant suspicions; and it may be presumed that every exertion would have been made to supply the deficiency. Belzoni and others discovered rudely manufactured teeth in the sarcophagæ of the Egyptians.

Again, as regards the use of gold leaf, Sir Gardiner Wilkinson states as a singular fact, that the Egyptians stopped teeth with gold. Proof of this was obtained by the examination of some mummies from Thebes. Bridgework was not unknown to the ancients. Dr. George Evans gives some illustrations of their primitive character and antique methods. We also have historical evidence that the general appearance of the teeth and their diseases, attracted considerable attention among the Greeks and Romans. The wearing of artificial teeth formed the subject of satire of some of their poets. Hippocrates and Galen mention sundry electuaries for beautifying the teeth.

The subdivision of medical practice to which I referred before, justifies us in concluding that dentistry engaged as large a share of the attention of the ancients as did any other branch of the healing art. Albucases, an Arabian physician, who lived in the early part of the eleventh century, wrote on diseases of the teeth and gave drawings of instruments then in use for extracting, scraping and other dental operations. He directs that if hollow, they should be stopped with cotton, refers to filing teeth and fastening loose ones with gold thread.

However, giving the ancients the credit to which they are entitled, it is not at all extraordinary that some of their opinions and methods should be useless for our purposes. They did not have the aid of that mighty power, the microscope; at least no mention is made of microscopic investigations in those times to which I have been alluding.

It is somewhat difficult to appreciate the observations of Hippocrates, who describes the teeth as glutinous extracts, from which the fatty matter has been burnt out by heat, and who affirms that they are harder than the other bones because they have no heat in them.

Aristotle, who has some observations respecting the teeth of man and animals, declares them to be the only bones which grow through the whole life, saying that if they did not they would soon be worn away by attrition. He adds that the growth is manifested in those teeth which have lost their corresponding opposite in the other jaw, referring of course to the elongation of teeth arising from the want of opposing force.

Now, by these observations from ancient authorities, I think I have shown the antiquity of our calling, and the attention bestowed upon the teeth at the earliest period of medical history.

I shall, however, proceed to bring my observations somewhat nearer to our own time. About three hundred years ago, the dental art began to receive that peculiar attention to which, from its importance and general usefulness, it was entitled.

About that time there were a number of treatises published on the subject, and while their usefulness has been greatly diminished by discoveries since made, still they are interesting as evidence that dental surgery was in the sixteenth century considered of very great importance, and that time and experience only were required to raise it to its proper station in medical art. Fouchard, of the eighteenth century, was the first to classify the diseases of the teeth. Previous to his time the practitioners appear to have merely considered the teeth in their mechanical arrangement taking little or no account of them as complex organic structures, entering by their own vitality into the formation of the living body.

Fouchard not only directed attention to the construction and separate treatment of the teeth, but he also pointed out the indications which, in common with the adjacent parts, they furnish of the general state of the health. It was without question the most important advancement in dental science to demonstrate that the teeth afford an indication, not merely of the apparent, but also of the innate and fundamental constitution of individuals.

It was, however, in 1747 that Haller, after thirty-six years of close study, published his remarkable work on physiological and pathological science, the results of actual observation of the laws that govern the growth and decay of living bodies. The first work that appeared in English in a popular form was by Berdmore, and was published in 1770. In 1772 the celebrated work on the "Natural History of the Teeth," by the great John Hunter was published. This was followed by a dissertation on the "Structure of the Teeth of Man and Animals," by Robert Blake. So much had the subject grown in consideration that by the end of the eighteenth century more than one hundred works had been published on the subject, including those of Malphigi, Purkinge, Retzius, Muller, &c.

Time will not permit me on this occasion to enumerate the various valuable contributions to dental science that have since been published; but I may, without a desire to lessen the merits of others, mention the names of Fox, Bell, Goodsir, Nasmyth, Tomes, Owen, Magitot, Wedl, Harris, Salter, Kingsly, Garretson, Richardson, Taft, Black, Miller and a host of others. Men identified with our profession and who stand pre-eminent as representatives of dental science and art.

In conclusion permit me to say that while the present is an age compulsive of deep thought and earnest reflections—an age that lays manly hold on things now that were once only dreamed of and hoped for; par excellence, an age of progress and scientific discoveries.

I would not be acting in accordance with my own impulse if I failed to congratulate the members of the Northern Ohio Dental Association for what they have done by associated effort in elevating the standard of dental science and art. I said by associated effort. That is in harmony with a law of the universe: "It is not good for man to be alone," means something. True, some of the pioneer members, men of broad minds and warm hearts, have passed out through the silence into the beyond, yet their influence still lives, and of them we say, All hail! Well done!

Congratulate you because today your chosen profession stands the peer of any of the learned professions.

Thanking you one and all for the uniform courtesy shown me while acting as your presiding officer and soliciting a like indulgence for my worthy successor my task is ended.

---

Dr. E. G. Tucker exhibited before the Society of the American Academy of Dental Science a case of crown and bridgework that he had inserted in a ladie's mouth in 1844, and which was worn with satisfaction forty-five years, until 1889. The teeth were porcelain, carved, single teeth (left upper central incisor and right and left upper canines) set on a gold plate, with gold pivots attached, and the three gold pivots were inserted into the hickory pivots which had previously been placed in the roots.

